# Kisspeptin neuron projections to oxytocin neurons are not necessary for parturition in the mouse

**DOI:** 10.1007/s00429-023-02670-7

**Published:** 2023-06-30

**Authors:** Shalini S. Kumar, Gregory T. Bouwer, Meliame K. Jackson, Michael R. Perkinson, Fiona J. McDonald, Colin H. Brown, Rachael A. Augustine

**Affiliations:** 1 Brain Health Research Centre, University of Otago, Dunedin, New Zealand; 2 Centre for Neuroendocrinology, University of Otago, Dunedin, New Zealand; 3 Department of Physiology, School of Biomedical Sciences, University of Otago, PO Box 56, Dunedin, 9054 New Zealand

**Keywords:** Oxytocin, Kisspeptin, Paraventricular nucleus, Supraoptic nucleus, Periventricular nucleus, Parturition

## Abstract

Oxytocin is synthesized by hypothalamic supraoptic nucleus (SON) and paraventricular nucleus (PVN) neurons and is released from the posterior pituitary gland to trigger uterine contractions during parturition. In rats, oxytocin neuron innervation by periventricular nucleus (PeN) kisspeptin neurons increases over pregnancy and intra-SON kisspeptin administration excites oxytocin neurons only in late pregnancy. To test the hypothesis that kisspeptin neurons excite oxytocin neurons to trigger uterine contractions during birth in C57/B6J mice, double-label immunohistochemistry for kisspeptin and oxytocin first confirmed that kisspeptin neurons project to the SON and PVN. Furthermore, kisspeptin fibers expressed synaptophysin and formed close appositions with oxytocin neurons in the mouse SON and PVN before and during pregnancy. Stereotaxic viral delivery of caspase-3 into the AVPV/PeN of Kiss-Cre mice before mating reduced kisspeptin expression in the AVPV, PeN, SON and PVN by > 90% but did not affect the duration of pregnancy or the timing of delivery of each pup during parturition. Therefore, it appears that AVPV/PeN kisspeptin neuron projections to oxytocin neurons are not necessary for parturition in the mouse.

## Introduction

Oxytocin is synthesized in magnocellular neurons of the hypothalamic supraoptic nucleus (SON) and paraventricular nucleus (PVN) and its release into the circulation from the posterior pituitary gland is increased during parturition to trigger uterine contractions (Brown 2016). While oxytocin is not essential for parturition (Nishimori et al. 1996) as oxytocin receptor knock-out mice still give birth, it is required for normal parturition because parturition is delayed and prolonged in rats by oxytocin receptor antagonism (Antonijevic et al. 1995). While increased oxytocin neuron activation during parturition is relayed via activation of brainstem noradrenergic afferents by cervical stretch receptors (Meddle et al. 2000; Douglas et al. 2001), central noradrenergic receptor activation is not sufficient to trigger parturition in late-pregnant rats (Lipschitz et al. 2004). Hence, it appears that other mechanisms must also contribute to oxytocin neuron activation during parturition.

Kisspeptin is the protein product of the *Kiss1* gene and is the endogenous ligand for the kisspeptin receptor (Kiss1R, GPR54; Kotani et al. 2001). In rodents, kisspeptin neurons are found in three hypothalamic areas: the anteroventral periventricular nucleus (AVPV), periventricular nucleus (PeN) and arcuate nucleus (ARC; Clarkson et al. 2009; Herbison 2008), and project widely across the brain, including to the SON and PVN (Desroziers et al. 2010).

In rats, kisspeptin neuron projections to the SON originate from the PeN and kisspeptin fiber density around the SON is maximal in late pregnancy (Seymour et al. 2017; Augustine et al. 2018). While intravenous kisspeptin excites rat oxytocin neurons across pregnancy and lactation by activation of vagal afferents (Scott and Brown 2011, 2013), central kisspeptin excitation of rat oxytocin neurons emerges over pregnancy to a maximum in late pregnancy (Augustine et al. 2018), and is mediated by a local action within the SON (Abbasi et al. 2022b) that does not involve activation of the canonical kisspeptin signaling pathway in oxytocin neurons (Abbasi et al. 2022a). Hence, it appears that kisspeptin neurons specifically excite oxytocin neurons in late-pregnant rats. Therefore, to test the hypothesis that kisspeptin neuron projections to oxytocin neurons are necessary for normal parturition, we first moved to a C57/B6J mouse model to establish the neuroanatomy of kisspeptin projections to the mouse SON and PVN, and to investigate whether there was any plasticity in the system during pregnancy using immunohistochemistry. Second, adeno-associated virus (AAV) was injected into the AVPV/PeN of kisspeptin-internal ribosome entry site-Cre (Kiss-Cre) mice to trigger caspase-induced cell death in kisspeptin neurons, to cause a loss in kisspeptin fiber innervation to the SON/PVN and thereby prevent oxytocin neuron activation by kisspeptin in late pregnancy. AVPV/PeN kisspeptin neuron ablation resulted in near total loss of kisspeptin immunoreactivity in the SON and PVN but did not affect parturition, suggesting that these projections to oxytocin neurons are not necessary for parturition in mice.

## Materials and methods

### Ethical approval

All experimental procedures were approved by the University of Otago Animal Ethics Committee and were carried out in accordance with the New Zealand Animal Welfare Act (1999) and associated guidelines.

### Animals

Female adult C57/B6J, Kiss-Cre^+^ and Kiss-Cre^−^ mice (de Croft et al. 2012) were group-housed in the University of Otago animal facility under controlled conditions (12 h light, 12 h dark cycle: lights on at 0600 h, 22 ± 1 °C) with free access to food and water. Mice had their estrous cycles monitored daily using vaginal cytology to examine the appearance of the epithelial cells. On proestrus, females were placed overnight in a cage with a male. Mating was considered to be successful when a vaginal plug was visualized on the following morning and this was recorded as day 1 of gestation (G1). For mice that gave birth, the day of birth was recorded as day 1 post-partum (PP1).

### Kisspeptin and oxytocin fluorescent immunohistochemistry in the supraoptic and paraventricular nuclei

Mice were anesthetized with sodium pentobarbitone (10 mg kg^−1^, IP) and transcardially perfused with 25 ml of 4% paraformaldehyde (PFA). The brains were removed, post-fixed in 4% PFA for 1 h and then in 30% sucrose/0.1 M phosphate buffer for 24–48 h, and frozen. Three series of 30 µm coronal sections were cut on a freezing microtome. Fluorescent immunohistochemistry for kisspeptin and oxytocin in the SON/PVN involved incubating sections from non-pregnant (NP, no specific day of the estrous cycle), day 7 (G7), 14 (G14) and 19 pregnant (G19) and day 7 lactating (PP7) C57/B6J mice in a cocktail of polyclonal rabbit anti-kisspeptin-10 (1:25,000, AC 564, A. Caraty, INRA, France, RRID:AB_2622231; Franceschini et al. 2006) and monoclonal mouse anti-oxytocin antiserum (1:5000, MAB5296 Millipore, MA, USA, RRID:AB_2157626; Dabrowska et al. 2011) in incubation solution (Tris-buffered saline (TBS) containing 0.3% Triton X-100, 0.25% bovine serum and 2% normal goat serum) for 48 h at 4 °C on an orbital shaker. Sections were then incubated in a cocktail of Alexa Fluor goat anti-rabbit 568 (1:500, A11036, Molecular Probes, OR, USA) and Alexa Fluor goat anti-mouse 488 (1:500, A11029, Molecular Probes, OR, USA) in incubation solution for 90 min. Sections were mounted onto gelatin-coated slides and cover-slipped using Vectashield Hardset mounting medium (H-1400, Vector Laboratories, Burlingame, CA, USA).

Z-series stacks (one section per area per mouse) were captured at 0.5 µm intervals through the SON and PVN using a Nikon A1R multiphoton confocal microscope under a 40 × oil-immersion objective and three-dimensional images were reconstructed using Nikon NIS Elements AR imaging software (Nikon Instruments Inc. Melville, NY, USA). More specific areas were also scanned using boxes of fixed-size (106 μm × 26.5 μm) in the SON (above the optic chiasm, where oxytocin neuron cell bodies predominantly reside (Pirnik et al. 2004)) and PVN; one box medially next to the third ventricle (the parvocellular division of the PVN), and the other 150 µm more lateral to the third ventricle, to incorporate the magnocellular division of the PVN (− 0.82 mm posterior from Bregma, based on the cytoarchitecture as described previously (Biag et al. 2012)). The fraction of kisspeptin signal-containing voxels to total voxels in the region of interest was calculated using IMARIS Image Analysis Software 9.8 (Oxford Instruments, Zurich, Switzerland) and expressed as a percentage of the area counted. The regions of interest were also analyzed for close appositions between kisspeptin fibers and oxytocin neurons using the spot counter in IMARIS. A surface was applied to the oxytocin neurons and spots were created for the kisspeptin immuno-positive fibers. Close appositions were defined as any spot from kisspeptin fibers contacting the surface of an oxytocin cell body or proximal dendrite. The number of oxytocin neurons within each box were counted in each area (SON; 2.58 ± 0.41) and did not differ between box 1 and box 2 in the PVN (3.64 ± 0.43 and 4.88 ± 0.48, respectively). Kisspeptin fiber density and appositions did not differ between box 1 and box 2 either. Therefore, the results were averaged for presentation.

### Kisspeptin and synaptophysin fluorescent immunohistochemistry in the supraoptic and paraventricular nuclei

Double-label fluorescent immunohistochemistry for kisspeptin and synaptophysin in the SON and PVN sections was carried out as described above, except sections were incubated in a cocktail of polyclonal rabbit anti-kisspeptin-10 (1:25,000, AC 564, A. Caraty, INRA, France, RRID:AB_2622231) and mouse anti-synaptophysin antiserum (1:1000, MAB368, Millipore, RRID:AB_94947; Yamanaka et al. 2011) for five nights at 4ºC. Z-series stacks were photographed through the SON and PVN at 1 µm intervals using a 60 × objective on a Nikon A1R confocal microscope and three-dimensional images were reconstructed using Nikon NIS Elements AR. The percentage of synaptophysin on kisspeptin fibers in the regions of interest were calculated as a percentage of co-localization using IMARIS. There was no remaining SON tissue from G7 mice to include in this part of the study.

### Viral injections to ablate the anteroventral periventricular and periventricular nuclei kisspeptin neurons

Caspase-induced cell death was targeted to kisspeptin neurons in Kiss-Cre mice by stereotaxic injection into the APVP/PeN at least five days (range: 5–14 days) before mating. This timeline was chosen to allow full recovery from the effects of surgery before mating and because pilot experiments (data not shown) revealed that between two and four weeks was required for maximal kisspeptin neuron ablation. Kiss-Cre^+^ and Kiss-Cre^‒^ mice were anesthetized with 4.5% isoflurane, placed in a stereotaxic apparatus and anesthesia was maintained at 2% isoflurane. Mice were given simultaneous bilateral injections (using 1 µL Hamilton syringes, Hamilton Company, NV, USA) of Cre-dependent caspase-3 delivered through the AAV5-flex-taCasp3-TEVp virus or AAV5-flex-C3-Tp (Vector Core, University of North Carolina; 1 µL/side of brain) system into the AVPV/PeN (0.7 mm anterior, 0.3 mm lateral and 5.7 mm ventral to bregma (Paxinos and Watson 2007)). The syringes were left in situ for 3 min before and 10 min after the injections. Mice were then allowed to recover for at least five days post injection. Estrous cycles were monitored daily from the day of injection. For those mice that displayed a first proestrus within the 5 days post injection recovery period, cycle length was determined by the number of days between the first two proestrous days following injection. Timed mating with a male occurred on the first proestrus, after the recovery period, to determine the duration of pregnancy in each mouse. Female mice were checked for the presence of a vaginal plug, which denoted day 1 of gestation. Mice were smeared for 14 days post-mating and pregnancy was confirmed by the presence of continual diestrous smears.

### Video recording of mouse parturition

On G14, Kiss-Cre^+^ and Kiss-Cre^‒^ mice were moved to a separate room dedicated to video recording for acclimatization to the new environment. Two cameras (Sony Chip Wired Bird Box Camera, Green Feathers, Bristol, UK) with infra-red night vision were installed on either side of each cage to record parturition. Cameras were connected to a desktop computer enabled with the software, iSpy 64-bit CCTV Recorder, which recorded continuously with tracked time and date. Dams were monitored continuously from G17 until PP4. The dams were randomized, coded and underwent blind analysis, with the experimenter being unaware of the groups. The precise timing of birth was determined from the video recording by the appearance and complete delivery of the first pup, and gestation length (days) was calculated. After analysis of all the data, the results were decoded, grouped, and expressed as mean ± SEM. Total parturition duration (min) was determined as the time between the complete delivery of the first pup to the complete delivery of the last pup. Mean time (min) of birth between each pup was determined as the time between the complete delivery of consecutive pups. Total number of live pups were counted at the end of parturition and each day thereafter. Four days post-partum (or 28 days post-caspase injection in mice that did not get pregnant), mice were anesthetized with sodium pentobarbitone (10 mg kg^−1^, IP), transcardially perfused and the brains removed, frozen and sliced on a freezing microtome, as described above.

### Kisspeptin immunohistochemistry in the anteroventral periventricular, periventricular, supraoptic, paraventricular and the arcuate nuclei of Kiss-cre^±^ mice

Free-floating chromogen immunohistochemistry for kisspeptin was undertaken as previously reported (Clarkson and Herbison 2006; Hellier et al. 2018) to evaluate and confirm the presence or absence of kisspeptin immunoreactivity in the AVPV/PeN after caspase-3 ablation in Kiss-Cre mice. Sections were incubated in polyclonal rabbit anti-kisspeptin-10 antibody (1:25,000; AC 564, A. Caraty, INRA, France, RRID:AB_2622231; Franceschini et al. 2006) for 48 h at 4 °C. Sections were then incubated in biotinylated goat anti-rabbit IgG (1:200; ZA0520, Vector Laboratories, Burlingame, CA, USA) for 90 min at room temperature, followed by incubation in Vectastain Elite ABC (Vector Laboratories, Burlingame, CA, USA). Sections were incubated in 3,3’-diaminobenzidine (DAB Peroxidase (HRP) Substrate Kit, Vector Laboratories) with nickel enhancement that resulted in a black precipitate and then mounted onto gelatin-coated slides and cover-slipped with Fluoromount-G™ mounting medium (00-4958-02, Invitrogen, USA).

To determine whether the number of kisspeptin-positive neurons in the AVPV/PeN differed between the Kiss-Cre^+^ pregnant and non-pregnant mice and Kiss-Cre^‒^ groups, sections were visualized under an Olympus BX61 light microscope and photographed at 10 × objective using QImaging Micropublisher 5.0 RTV camera (QImaging, Canada). Numbers of kisspeptin-positive soma were counted in three sections per mouse and the mean value for each group calculated. Data were analyzed as mean number of kisspeptin neurons ± SEM.

### Kisspeptin fluorescent immunohistochemistry in the supraoptic, paraventricular and arcuate nuclei

Single-label fluorescent immunohistochemistry for kisspeptin in the SON, PVN and ARC sections was carried out as previously described (Seymour et al. 2017). Briefly, every second or third section from the SON, PVN or ARC of each mouse brain was immunolabeled with anti-kisspeptin-10 antibody (1:25,000; AC 564, A. Caraty, INRA, France, RRID:AB_2622231; Franceschini et al. 2006) and secondary antibody Alexa Fluor goat anti-rabbit 568 (1:500, A11036, Molecular Probes, USA), respectively.

To determine whether the density of kisspeptin-positive fibers differed between the Kiss-Cre^+^ pregnant and non-pregnant mice and Kiss-Cre^‒^ groups, images from the SON, PVN and ARC were acquired using an inverted confocal microscope (Nikon A1R Inverted Confocal Laser Scanning Microscope, Nikon Instruments Inc, Tokyo, Japan) in Z-series stacks. A Z-series of 15–17 stacks was captured using excitation at 488 nm and 561 nm, 20 × objective, a pinhole of 4 µm and a slice interval of 1.65 µm at dimensions 1024 μm × 1024 μm. Fiber density was measured as previously described (Patisaul et al. 2008). Using Image J (NIH), a sub stack of images was created to control for section thickness throughout the samples. Images were then binarized to a threshold to allow best signal-to-noise ratio and skeletonized to make the fibers one pixel thick for fiber thickness normalization. The voxel counter plugin (NIH) was then used to count the number of voxels. The fraction of signal-containing voxels to total voxels in each region was calculated, and the mean value for each group was calculated. Data were analyzed as mean fiber density in % ± SEM of three sections per mouse.

### Statistical analysis

All data were analyzed using GraphPad Prism (Version 8.00). Values are expressed as mean ± SEM unless stated otherwise. Two-tailed unpaired Student’s *t*-tests were used to compare between two groups and one-way ANOVA was used to compare between three or more groups where *n* ≥ 5; where the *F*-ratio was significant, all-pairwise Dunnett’s post hoc tests were applied. Where *n* < 5, non-parametric Mann–Whitney tests were used to compare between two groups and Kruskal–Wallis ANOVA on ranks was used to compare between three or more groups; where the ANOVA on ranks was significant, all-pairwise Dunn’s post hoc tests were applied.* P* ≤ 0.05 was considered significant.

## Results

### Kisspeptin neurons project to the supraoptic and paraventricular nuclei

**Fig. 1:**
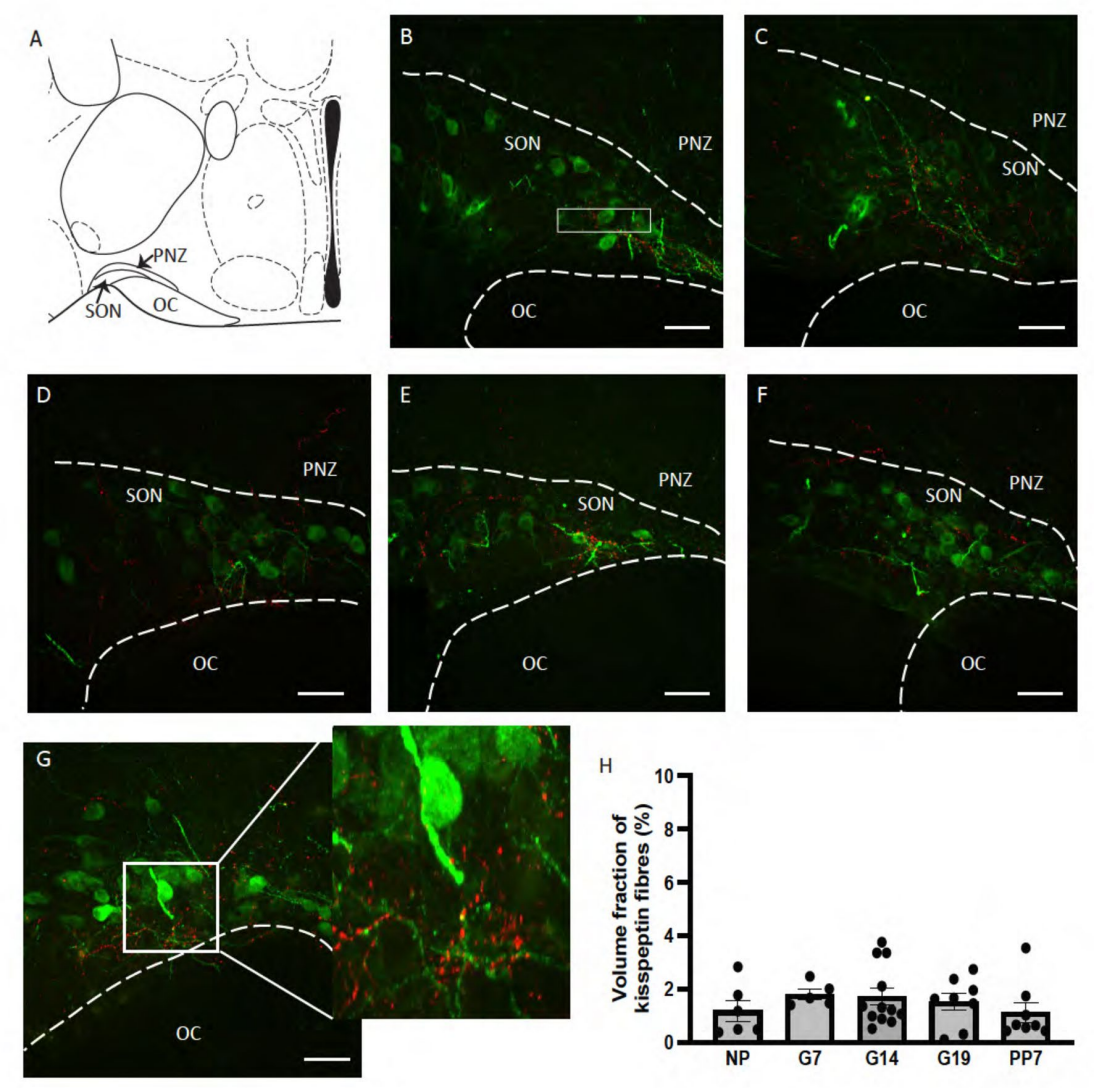
Kisspeptin fibers in the supraoptic nucleus in non-pregnant, pregnant and lactating mice. **A** Schematic representation of a coronal section through the mouse brain, showing the location of the supraoptic nucleus (SON). **B–F** Representative confocal images (0.7 mm–0.8 mm posterior to Bregma) of double-label immunohistochemistry for oxytocin (green) and kisspeptin (red) in the SON of non-pregnant (NP; **B**), day 7 pregnant (G7; **C**), day 14 pregnant (G14; **D**), day 19 pregnant (G19; **E**) and day 7 lactating (PP7; **F**) mice. **G** Representative image of kisspeptin fibers bundling around oxytocin fibers in the SON. **H** Mean volume fraction of kisspeptin labeling in the SON of NP, G7, G14, G19 and PP7 mice. There was no significant difference in the density of SON kisspeptin labeling between the groups (*F*_4,34_ = 0.69, *P* = 0.60, one-way ANOVA). The box within the SON (**B**) represents the approximate location that high magnification Z-stacks were taken for fiber density and apposition analysis. Scale bars = 50 µm. *OC* optic chiasm, *PNZ* perinuclear zone

**Fig. 2:**
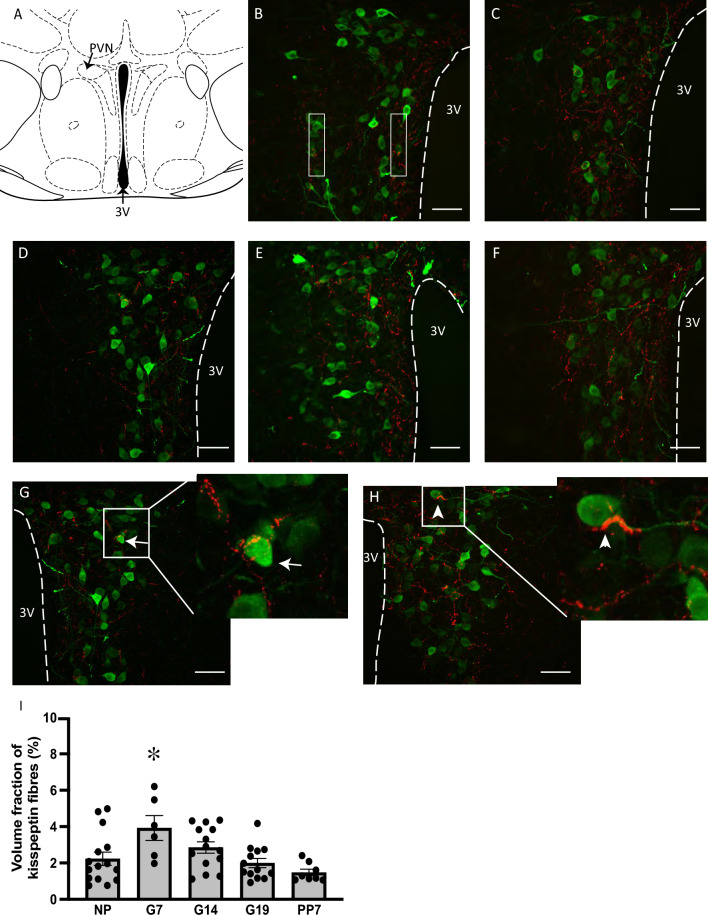
Kisspeptin fibers in the paraventricular nucleus in non-pregnant, pregnant and lactating mice. **A** Schematic representation of a coronal section through the mouse brain, showing the location of the paraventricular nucleus (PVN). **B–F** Representative confocal images (0.7 mm–0.8 mm posterior to Bregma) of double-label immunohistochemistry for oxytocin (green) and kisspeptin (red) in the PVN of non-pregnant (NP; **B**), day 7 pregnant (G7; **C**), day 14 pregnant (G14; **D**), day 19 pregnant (G19; **E**) and day 7 lactating (PP7; **F**) mice. **G**, **H** Representative images of kisspeptin fibers bundling around an oxytocin cell body (**G**; arrows) and proximal dendrite (**H**; arrowheads) in the PVN. **I** Mean volume fraction of kisspeptin labeling in the PVN of NP, G7, G14, G19 and PP7 mice. There was a significant difference in the density of PVN kisspeptin labeling between the groups (*F*_4,51_ = 4.68, *P* = 0.003, one-way ANOVA), with significantly higher labeling in G7 mice than in NP mice (*P = 0.02, NP vs G7, Dunnett’s post hoc test). The boxes within the PVN (**B**) represent the approximate location that high magnification *Z*-stacks were taken for fiber density and apposition analysis. Scale bars = 50 µm. 3 V = third ventricle

Kisspeptin-positive fibers were observed among oxytocin-positive neurons throughout the rostral extent of the SON in NP, G7, G14, G19 and PP7 C57/B6J mice (Fig. 1B–F) and PVN (Fig. 2B–F). Some fibers were also evident in the perinuclear zone (PNZ), surrounding the SON, in all groups (Fig. 1B–F). Kisspeptin fibers bundled around oxytocin fibers and cell bodies in the SON (Fig. 1G) and PVN (Fig. 2G, H). The volume fraction of kisspeptin labeling was similar in the SON of all groups (Fig. 1H; *F*_4,34_ = 0.69, *P* = 0.60, one-way ANOVA). In the PVN, the volume fraction of kisspeptin labeling was different between groups (Fig. 2I, *F*4,51 = 4.68, *P* = 0.003, one-way ANOVA), with higher expression in G7 mice than in NP mice (*P* = 0.02, NP vs G7, Dunnett’s post hoc test).

### Kisspeptin forms appositions with oxytocin neurons in the supraoptic and paraventricular nuclei

**Fig. 3:**
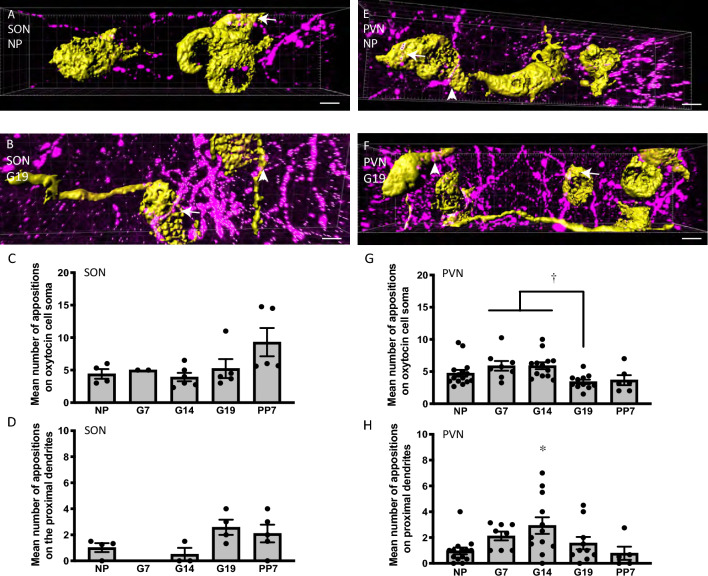
Kisspeptin appositions on oxytocin neurons in non-pregnant, pregnant and lactating mice. **A**–**D** 3D confocal *z*-series stacks of oxytocin (yellow) and kisspeptin (magenta) labeling in the supraoptic nucleus (SON; **A** and **B**) and paraventricular nucleus (PVN; **E** and **F**) from non-pregnant (NP; **A** and **E**) and day 19 pregnant (G19; **B** and **F**) mice. Appositions (white) can be seen between kisspeptin fibers and oxytocin cell bodies (arrows) and proximal dendrites (arrowheads). **C**, **D** and **G**, **H**. Mean number of appositions of kisspeptin fibers with oxytocin cell bodies in the SON (**C**) and PVN (**G**) and with proximal dendrites in the SON (**D**) and PVN (**H**). In the SON, there was no effect of reproductive status on the number of appositions on cell bodies (*H*_(2)_ = 7.96, *P* = 0.09) or proximal dendrites (*H*_(2)_ = 6.1, *P* = 0.098). **G** In the PVN, there was a significant effect of reproductive status on the number of appositions on cell bodies (*F*_4,48_ = 4.04, †*P* = 0.007, one-way ANOVA), although there was no significant difference between NP and any of the other groups (Dunnett’s post hoc tests). **H** There was higher number of appositions on the proximal dendrites of oxytocin neurons in the PVN of G14 mice than in NP mice (Fig. 3H, *F*_4,44_ = 3.46, *P = 0.015, one-way ANOVA, *P* = 0.007, Dunnett’s post hoc test). Scale bars = 7 µm

Kisspeptin fibers made few appositions with oxytocin neurons in the SON (Fig. 3A, B) and PVN of (Fig. 3E–F) C57/B6J mice. There were a similar number of appositions with oxytocin cell bodies (*H*_(2)_ = 7.96, *P* = 0.09) and proximal dendrites (*H*_(2)_ = 6.10, *P* = 0.10) in the SON in NP, G7, G14, G19 and PP7 mice (Fig. 3C, D). While there was a difference in the number of appositions with oxytocin cell bodies in the PVN (Fig. 3G, *F*_4,48_ = 4.04, *P* = 0.007), there was no difference between NP and any of the other groups, rather the difference was between G7 and G14 compared to G19 (Dunnett’s post hoc tests). There was a higher number of appositions on the proximal dendrites of oxytocin neurons in the PVN of G14 mice than in NP mice (Fig. 3H, *F*_4,44_ = 3.46, *P* = 0.015, one-way ANOVA, *P* = 0.007, Dunnett’s post hoc test).

### Synaptophysin expression in kisspeptin fibers in the supraoptic and paraventricular nuclei

**Fig. 4:**
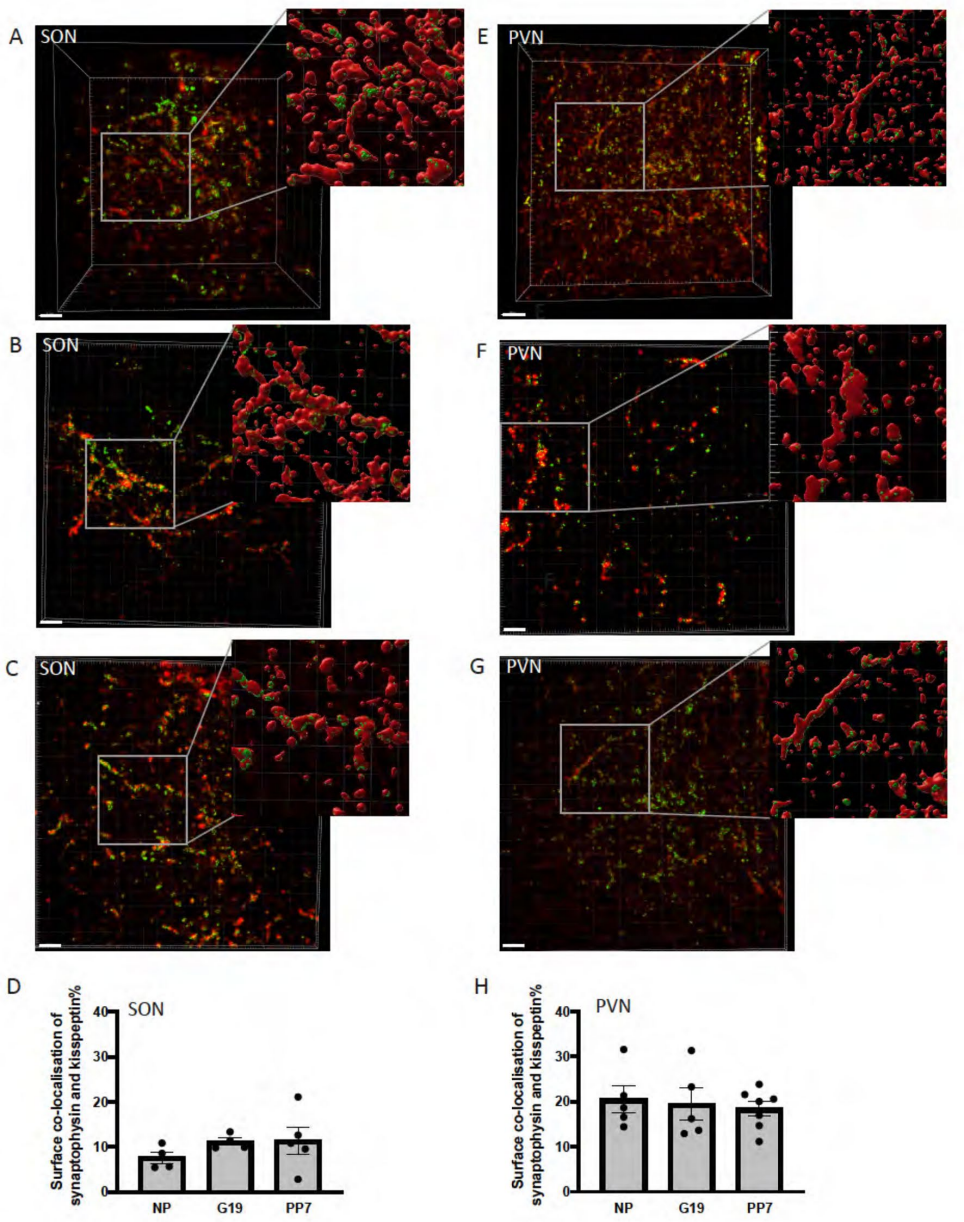
Synaptophysin expression in kisspeptin fibers in the supraoptic and paraventricular nuclei of non-pregnant, pregnant and lactating mice. **A**–**F** Representative confocal images of kisspeptin fibers (red) and synaptophysin (green), in the supraoptic nucleus (SON; **A**–**C**) and paraventricular nucleus (PVN; **E**–**G**) in non-pregnant (NP; **A**, **E**), day 19 pregnant (G19; **B**, **F**) and day 7 lactating (PP7; **C**, **G**) mice. **D**, **H** Mean surface area of kisspeptin fibers co-labeled with synaptophysin in the SON (**D**) and PVN (**H**) of NP, G19 and PP7 mice. There was no effect of reproductive status on co-localization in the SON (*H*_(2)_ = 2.58, *P* = 0.29) or PVN (*F*_2,14_ = 0.17, *P* = 0.84). Scale bars = 20 µm

There was a similar level of synaptophysin co-localization in kisspeptin fibers in both the SON (*H*_(2)_ = 2.58, *P* = 0.29) and PVN (*F*_2,14_ = 0.17, *P* = 0.84) in NP, G19 and PP7 C57/B6J mice (Fig. 4).

### Caspase-3 ablation of the anteroventral periventricular and periventricular nuclei kisspeptin neurons

**Fig. 5:**
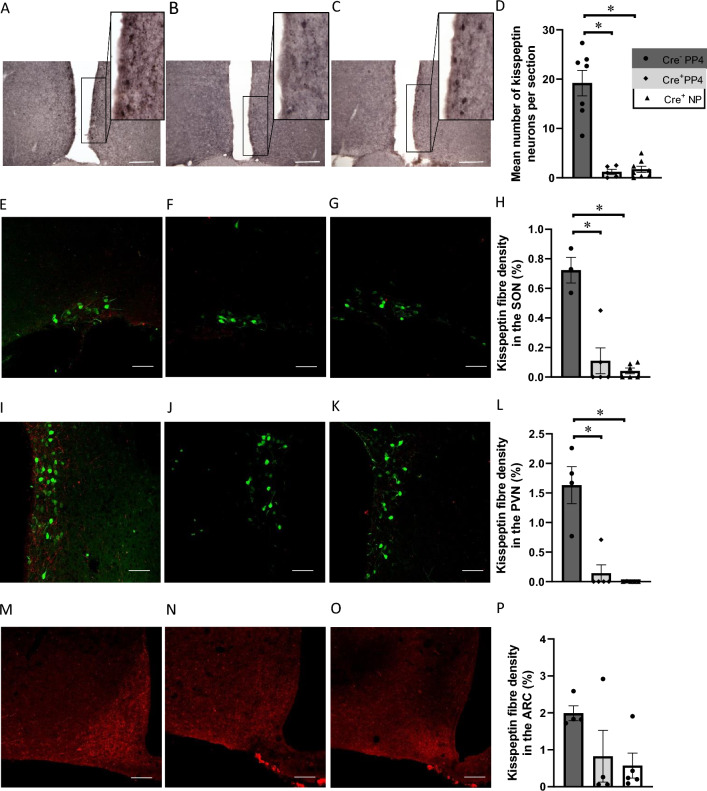
Quantification of kisspeptin labeling in the anteroventral periventricular and periventricular nuclei and fibers in the paraventricular and supraoptic nuclei after ablation. **A**–**C** Representative light microscopy images of the periventricular nucleus (PeN) in Kiss-Cre^‒^ post-partum day 4 (PP4) (**A**) and Kiss-Cre^+^ PP4 (**B**) and Kiss-Cre^+^ non-pregnant (NP) mice (**C**). Mice were injected with caspase-3 to ablate kisspeptin neurons in the AVPV/PeN. Note the decrease in kisspeptin-positive soma and fibers as seen in the higher magnification images of the boxed insets in Kiss-Cre^+^ (**B**, **C**) AVPV/PeN when compared to the Kiss-Cre^‒^ mouse section (**A**). **D** The mean number of kisspeptin neurons in the AVPV/PeN between the groups was different (*F*_2,17_ = 39.37, *P = 0.039). The mean maximal number of kisspeptin neurons per section in Kiss-Cre^‒^ mice on PP4 was 19.21 ± 2.6 (*n* = 7) while Kiss-Cre^+^ PP4 mice had less with 1.21 ± 0.51 neurons (*n* = 5, *P < 0.001, Dunnetts post hoc test) and the Kiss-Cre^+^ NP mice had 1.73 ± 0.61 neurons (*n* = 8, *P* < 0.001, Dunnetts post hoc test) per section. **E–P** Representative confocal images of kisspeptin-positive fibers in the SON, PVN and ARC in Kiss-Cre^‒^ PP4 (**E**, **I** and **M**) and Kiss-Cre^+^ PP4 (**F**, **J** and **N**) and Kiss-Cre^+^ NP mice (**G**, **K** and **O**). Viral ablation of kisspeptin cells in the AVPV/PeN resulted in a significant loss of kisspeptin fiber immunoreactivity in the SON and PVN. **E**–**H** In the SON, the mean density of kisspeptin fibers (%) was different between the groups (*H*_(2)_ = 7.19, *P* = 0.02) with Kiss-Cre^+^ PP4 mice having less fibers (0.11 ± 0.09, *n* = 5) compared to Kiss-Cre^‒^ PP4 mice (0.72 ± 0.09, *n* = 3; *P = 0.04, Dunn’s post hoc test). There was also less kisspeptin fibers in the Kiss-Cre^+^ NP mice (0.04 ± 0.02, *n* = 6) than in the Kiss-Cre^−^ PP4 mice (*P = 0.02, Dunn’s post hoc test) (**H**). **I**–**L** The fiber density (%) in the PVN was different between the groups (*H*_(2)_ = 9.82, *P* = 0.003) with less fibers in the Kiss-Cre^+^ PP4 mice (0.14 ± 0.14, *n* = 5) than in Kiss-Cre^‒^ PP4 (1.63 ± 0.31, *n* = 4) mice (*P = 0.01, Dunn’s post hoc test) (**L**). There were also less kisspeptin fibers in the Kiss-Cre^+^ NP mice (0.002 ± 0.002, *n* = 5) than in the Kiss-Cre^−^ PP4 mice (*P = 0.01, Dunn’s post hoc test) (**L**). **M**–**P** In the ARC, the mean density of kisspeptin fibers (%) was not different between the groups (*H*_(2)_ = 2.93, *P* = 0.24). Kiss-Cre^‒^ PP4 mice fiber density was 1.99 ± 0.202, (*n* = 4), Kiss-Cre^+^ PP4 mice fiber density was 0.825 ± 0.7, (*n* = 4) and in Kiss-Cre^+^ NP mice it was 0.574 ± 0.34, (*n* = 5) (P). Scale bars = 50 µm

Bilateral injections of Caspase-3 effectively ablated kisspeptin cell bodies in the AVPV/PeN in Kiss-Cre^+^ female mice but not Kiss-Cre^‒^ female mice (Fig. 5A–C). Fourteen Kiss-Cre^+^ mice had successful ablation of kisspeptin neurons on both sides of the AVPV/PeN. There was a difference in the number of kisspeptin neurons in the AVPV/PeN (Fig. 5D) between the groups (*F*_2,17_ = 39.37, *P* = 0.039, one-way ANOVA). The mean maximal number of kisspeptin neurons per section in Kiss-Cre^−^ mice on PP4 was higher than in Kiss-Cre^+^ PP4 mice (*P* < 0.001, Dunnetts post hoc test) and in Kiss-Cre^+^ NP mice (*P* < 0.001, Dunnetts post hoc test).

### Kisspeptin fiber density in the supraoptic, paraventricular and arcuate nuclei

Kisspeptin-positive fibers were observed in the SON, PVN and ARC in all groups of Kiss-Cre^±^ mice but a marked reduction was seen in both groups of Kiss-Cre^+^ mice after kisspeptin cell ablation in the AVPV/PeN. In the SON (Fig. 5E–G) the mean density of kisspeptin fibers (%) was different between the groups (*H*_(2)_ = 7.19, *P* = 0.02) with Kiss-Cre^+^ PP4 mice having less compared to Kiss-Cre^−^ PP4 mice (*P* = 0.04, Dunn’s post hoc test). There were also less kisspeptin fibers in the Kiss-Cre^+^ NP mice than in the Kiss-Cre^−^ PP4 mice (*P* = 0.02, Dunn’s post hoc test, Fig. 5H).

The fiber density (%) in the PVN (Fig. 5I–K) was different between the groups (*H*_(2)_ = 9.82, *P* = 0.003) with less fibers in the Kiss-Cre^+^ PP4 mice than in Kiss-Cre^−^ mice (*P* = 0.01, Dunn’s post hoc test). There were also less kisspeptin fibers in the Kiss-Cre^+^ NP mice than in the Kiss-Cre^−^ PP4 mice (*P* = 0.01, Dunn’s post hoc test, Fig. 5L).

In the ARC (Fig. 5M–P) the mean density of kisspeptin fibers (%) was not different between the groups (H_(2)_, *P* = 0.24, Fig. 5P).

### Anteroventral periventricular and periventricular nuclei kisspeptin neurons are not necessary for parturition

**Fig. 6:**
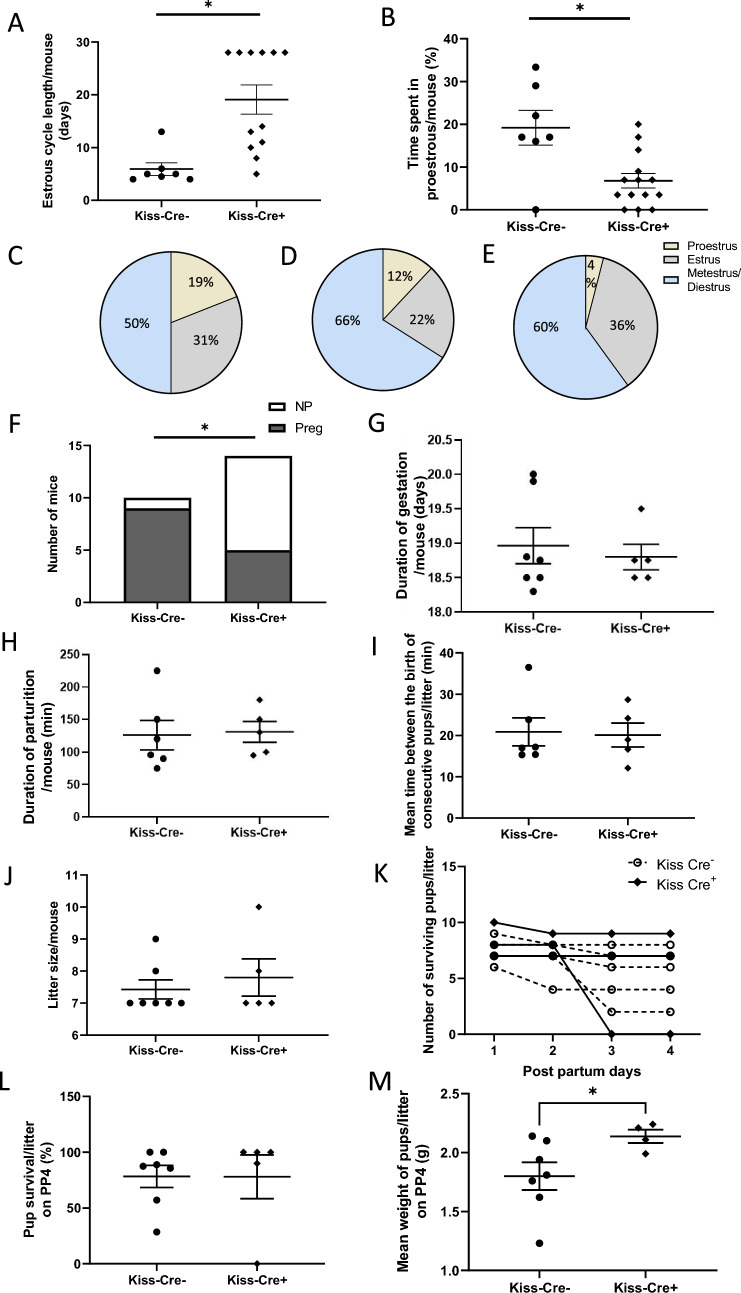
Caspase-3 ablation of anteroventral periventricular and periventricular nuclei kisspeptin neurons did not affect parturition. **A** The data show the mean ± SEM length of the estrous cycle between Kiss-Cre^‒^ (*n* = 7) and Kiss-Cre^+^ (*n* = 12) mice injected with caspase-3 in the AVPV/PeN to ablate kisspeptin neurons. Kiss-Cre^+^ mice had significantly longer estrous cycles compared with Kiss-Cre^‒^ mice (*P = 0.003, Student’s *t*-test). **B** The data show the mean % ± SEM of time spent in proestrus between caspase-3 injected Kiss-Cre^‒^ (*n* = 7) and Kiss-Cre^+^ (*n* = 12) mice. Kiss-Cre^+^ mice spent significantly less time in proestrus compared with Kiss-Cre^‒^ mice (*P = 0.003, Student’s *t*-test). **C** The data show the percentage of time Kiss-Cre^‒^ mice spent in each day of the cycle. **D** The data show the percentage of time spent in each day of the cycle in Kiss-Cre^+^ mice who got pregnant. **E** The data show the percentage of time spent in each day of the cycle in Kiss-Cre^+^ mice that did not get pregnant. **F** Graphical representation of the number of caspase-3 injected Kiss-Cre^−^ and Kiss-Cre^+^ mice that became pregnant or failed to get pregnant after kisspeptin neuron ablation in the anteroventral periventricular and periventricular nuclei (AVPV/PeN). The proportion of mice that failed to get pregnant was higher in Kiss-Cre^+^ group (*n* = 14, *P < 0.01, Chi-square test). Only one mouse out of ten failed to get pregnant in the Kiss-Cre^‒^ group. **G** The data show the mean ± SEM length of gestation between caspase-3 injected Kiss-Cre^‒^ (*n* = 7) and Kiss-Cre^+^ (*n* = 5) pregnant dams (*P* = 0.8, Student’s *t*-test). **H** The data show the mean ± SEM duration of parturition between Kiss-Cre^‒^ (*n* = 7) and Kiss-Cre^+^ (*n* = 5) pregnant dams (*P* = 0.8, Student’s *t*-test). **I** The data show the mean ± SEM time between the birth of consecutive pups in Kiss-Cre^‒^ (*n* = 7) and Kiss-Cre^+^ (*n* = 5) pregnant dams (*P* = 0.87, Student’s *t*-test). **J** The data represent mean ± SEM litter size between the Kiss-Cre^‒^ mice (*n* = 7) and Kiss-Cre^+^ mice (*n* = 5, *P* = 0.55, Student’s *t*-test). **K.** Survival plot representing the pup survival over the course of lactation (0–PP4); the data represent the mean ± SEM percent of pup survival between Kiss-Cre^‒^ mice (*n* = 7) and Kiss-Cre^+^ mice (*n* = 5). Solid circles denote both groups on the same point on the graph. **L** The data represent the mean ± SEM percent of pup survival on PP4 between Kiss-Cre^‒^ mice (*n* = 7) and Kiss-Cre^+^ mice (*n* = 5, *P* = 0.99, Student’s *t*-test). **M** The data represent the mean weight of pups (g) from Kiss-Cre^+^ (*n* = 4) and Kiss-Cre^‒^ mice (*n* = 7). The mean weight of pups born to Kiss-Cre^+^ mice was higher than those born to Kiss-Cre^−^ mice (*P = 0.04, Mann–Whitney test)

Ablation of the AVPV/PeN kisspeptin neurons in female Kiss-Cre^+^ mice disrupted the estrous cycle. Kiss-Cre^+^ mice had longer estrous cycles (Fig. 6A, *P* = 0.003, Student’s *t*-test) and less days in proestrus compared with Kiss-Cre^−^ mice (Fig. 6B, *P* = 0.003, Student’s *t*-test). Kiss-Cre^+^ mice that fell pregnant spent longer in proestrus than Kiss-Cre^+^ mice that did not fall pregnant (Fig. 6D–E). There was a large proportion of Kiss-Cre^+^ females (nine out of 14) that failed to get pregnant after bilateral injection with AAV5-flex-C3-Tp, while only one female (one out of ten) Kiss-Cre^‒^ failed to get pregnant after the stereotaxic injection (Fig. 6F, *P* < 0.01, Chi-square test). Hence, the ablation of the AVPV/PeN kisspeptin neurons in female Kiss-Cre^+^ mice also reduced their ability to get pregnant. There was no difference in the duration of gestation between Kiss-Cre^+^ and Kiss-Cre^−^ females (Fig. 6G, *P* = 0.8, Student’s *t*-test). There was no difference in the duration of parturition (min) between Kiss-Cre^+^ and Kiss-Cre^−^ dams (Fig. 6H, *P* = 0.8, Student’s *t*-test). There was also no difference in the time between delivery of each pup between the two groups (Fig. 6I, *P* = 0.87, Student’s *t*-test). There was no difference in the mean litter size on PP1 between Kiss-Cre^+^ and Kiss-Cre^−^ dams (Fig. 6J, *P* = 0.55, Student’s *t*-test). Caspase-3 ablation of AVPV/PeN kisspeptin neurons did not affect the number of pups surviving in each Kiss-Cre^+^ litter when compared to Kiss-Cre^−^ litters (Fig. 6K). No difference was observed in the percent of pup survival between Kiss-Cre^+^ mice, and Kiss-Cre^−^ mice (Fig. 6L, *P* = 0.9, Student’s *t*-test). One Kiss-Cre^+^ dam lost all its pups after PP2 (0% survival), whereas no Kiss-Cre^−^ dams lost more than 50% of pups post-partum. The mean weight of pups born to the Kiss-Cre^+^ mice was higher on PP4 than the pups born to Kiss-Cre^−^ mice (Fig. 6M, *P* = 0.04, Mann–Whitney test).

## Discussion

Here, kisspeptin neurons have been shown to form close appositions with SON and PVN oxytocin neurons in non-pregnant mice and across pregnancy. Furthermore, kisspeptin fibers co-expressed synaptophysin in the SON and PVN, suggesting that kisspeptin might be locally released to excite oxytocin neurons in the mouse, as it is in the rat (Abbasi et al. 2022b). However, AVPV/PeN kisspeptin neuron ablation did not affect parturition in the mouse despite near total loss of kisspeptin fibers in the SON and PVN.

PVN (but not SON) kisspeptin fiber density was higher in early pregnancy than in late pregnancy, as was the number of kisspeptin fiber close appositions with PVN (but not SON) oxytocin neuron somata. Synaptophysin expression in SON and PVN kisspeptin fibers was similar between non-pregnant and pregnant mice. Whether synaptophysin expression is synaptic, or interactions between these neurons are mediated via volume transmission, as is the case for gonadotrophin-releasing hormone neurons (Liu et al. 2021) remains to be determined.

Ablating AVPV and PeN kisspeptin neurons with caspase almost entirely deleted kisspeptin fibers in the SON and PVN of Kiss-Cre^+^ mice. We cannot exclude the possibility that the ARC kisspeptin population projects to the AVPV/PeN and/or the SON/PVN (Pineda et al. 2016; Stincic et al. 2021), but our findings suggest that ARC kisspeptin innervation of the SON/PVN is likely negligible.

While there is plasticity in the kisspeptin projections to oxytocin neurons, this plasticity does not appear to be important for parturition because ablation of the projection did not affect parturition. Consistent with the well-characterized involvement of AVPV/PeN kisspeptin neurons in fertility (Clarkson et al. 2008), only five of 14 Kiss-Cre^+^ mice fell pregnant after mating. Estrous cycle length increased and the time Kiss-Cre^+^ mice spent in proestrus decreased, suggesting kisspeptin ablation disrupts the normal estrous cyclicity in mice. A large proportion of Kiss-Cre^+^ mice were acyclic and showed no or only one proestrous smear in the 28 days post-surgery, demonstrating that some AVPV/PeN kisspeptin neurons are necessary for the induction of proestrus and ovulation (Hu et al. 2015; Clarkson et al 2008; Piet et al. 2018). Kiss-Cre^+^ mice that fell pregnant spent longer in proestrous than Kiss-cre^+^ mice that did not fall pregnant. Presumably, sufficient ablation of AVPV/PeN kisspeptin neurons occurred in these mice to reduce fertility in less than two weeks, while functional ablation of AVPV/PeN kisspeptin neurons took longer in Kiss-Cre^+^ mice that fell pregnant. A similar impact on estrous cyclicity and fertility was seen in adult mice with complete kisspeptin neuron ablation (Mayer and Boehm 2011) although this did not discriminate between the AVPV/PeN and ARC subpopulations of kisspeptin cells.

Nevertheless, the numbers of AVPV/PeN kisspeptin neurons in Kiss-Cre^+^ mice four days after delivery was indistinguishable from that in Kiss-Cre^+^ mice that did not fall pregnant and was < 10% that of Kiss-Cre^−^ mice, suggesting that all Kiss-Cre^+^ mice were eventually impacted to the same degree. While it is impossible to exclude the possibility that the remaining kisspeptin projections to oxytocin neurons are sufficient to maintain normal parturition, it seems unlikely that so few kisspeptin neurons could sufficiently excite thousands of oxytocin neurons to trigger uterine contractions when reduced to similar numbers that cannot sustain fertility. Hence, the most likely explanation is that endogenous kisspeptin activation of oxytocin neurons may not be necessary to trigger uterine contractions in the mouse.

While ablation of AVPV/PeN kisspeptin neurons did not affect parturition, it is possible that the plasticity in the AVPV/PeN kisspeptin neuron projections to oxytocin neurons is involved in other aspects of pregnancy and mothering. Disruption of central oxytocin signaling impairs maternal behavior (Sanson and Bosch 2022). However, no interventions were made to directly investigate whether AVPV/PeN kisspeptin neuron ablation impacted maternal behavior because kisspeptin is involved in stress responses (McCosh et al. 2019). Nevertheless, there was no impact of AVPV/PeN kisspeptin neuron ablation on pup survival, and qualitative evaluation of video recordings did not suggest any obvious impairment in maternal behavior. If anything, lactation was enhanced in the dams with kisspeptin neuron ablation because their pups weighed more on day 4 of lactation than the pups from Kiss-Cre^−^ mice. Therefore, the function of the AVPV/PeN kisspeptin neuron projections to oxytocin neurons remains to be determined.

Kisspeptin projections to oxytocin neurons were investigated in mice because observations from rats suggested that this afferent input might be important for parturition. While there were broad similarities between species, there were also some differences. AVPV/PeN kisspeptin neuron projections were principally to the SON (and PVN) in mice but rat PeN kisspeptin neuron projections are principally to the PNZ surrounding the SON (Seymour et al. 2017). The PNZ is a rich source of glutamatergic and GABAergic innervation of the SON and PVN that relay information from many other brain areas (Brown et al. 2013). Kisspeptin was evident in the AVPV and PeN of non-pregnant mice as has been previously reported (Gottsch et al. 2004; Clarkson and Herbison 2006; Kauffman et al. 2007) but are almost absent from the AVPV and PeN of non-pregnant rats (Seymour et al. 2017). It remains to be determined whether species differences extend to the functional impact of AVPV and PeN kisspeptin neurons on parturition and, if so, which species is the better model for human pregnancy.

Taken together, our results suggest that there is plasticity in the AVPV/PeN projection to oxytocin neurons over pregnancy in mice, but this plasticity does not appear to be important for parturition, and its functional significance remains to be determined.

## Data Availability

All data generated or analyzed during this study are included in this published article.
